# A Serological Snapshot of COVID-19 Initial Stages in Israel by a 6-Plex Antigen Array

**DOI:** 10.1128/Spectrum.00870-21

**Published:** 2021-10-06

**Authors:** Morly Fisher, Haim Levy, Ella Fatelevich, Yafa Afrimov, Amir Ben-Shmuel, Ronit Rosenfeld, Tal Noy-Porat, Itai Glinert, Assa Sittner, Asaf Biber, Ana Belkin, Elad Bar-David, Reut Puni, Itzchak Levy, Ohad Mazor, Shay Weiss, Adva Mechaly

**Affiliations:** a Department of Infectious Diseases, Israel Institute for Biological Research, Ness-Ziona, Israel; b Department of Biochemistry and Molecular Genetics, Israel Institute for Biological Research, Ness-Ziona, Israel; c Sheba Medical Center and the Sackler Medical School, Tel Aviv University, Tel Aviv-Yafo, Israel; University of Arizona/Banner Health

**Keywords:** SARS-CoV-2, spike, nucleocapsid, COVID-19, Israel, serology, microarrays, multiplex

## Abstract

The first case of SARS-CoV-2 was discovered in Israel in late February 2020. Three major outbreaks followed, resulting in over 800,000 cases and over 6,000 deaths by April 2021. Our aim was characterization of a serological snapshot of Israeli patients and healthy adults in the early months of the COVID-19 pandemic. Sera from 55 symptomatic COVID-19 patients and 146 healthy subjects (early-pandemic, reverse transcription-quantitative PCR [qRT-PCR]-negative), collected in Israel between March and April 2020, were screened for SARS-CoV-2-specific IgG, IgM, and IgA antibodies, using a 6-plex antigen microarray presenting the whole inactivated virus and five viral antigens: a stabilized version of the spike ectodomain (S2P), spike subunit 1 (S1), receptor-binding-domain (RBD), N-terminal-domain (NTD), and nucleocapsid (NC). COVID-19 patients, 4 to 40 days post symptom onset, presented specific IgG to all of the viral antigens (6/6) in 54 of the 55 samples (98% sensitivity). Specific IgM and IgA antibodies for all six antigens were detected in only 10% (5/55) and 4% (2/55) of the patients, respectively, suggesting that specific IgG is a superior serological marker for COVID-19. None of the qRT-PCR-negative sera reacted with all six viral antigens (100% specificity), and 48% (70/146) were negative throughout the panel. Our findings confirm a low seroprevalence of anti-SARS-CoV-2 antibodies in the Israeli adult population prior to the COVID-19 outbreak. We further suggest that the presence of low-level cross-reacting antibodies in naive individuals calls for a combined, multiantigen analysis for accurate discrimination between naive and exposed individuals.

**IMPORTANCE** A 6-plex protein array presenting the whole inactivated virus and five nucleocapsid and spike-derived SARS-CoV-2 antigens was used to generate a serological snapshot of SARS-CoV-2 seroprevalence and seroconversion in Israel in the early months of the pandemic. Our findings confirm a very low seroprevalence of anti-SARS-CoV-2 antibodies in the Israeli adult population. We further propose that the presence of low-level nonspecific antibodies in naive individuals calls for a combined, multiantigen analysis for accurate discrimination between naive and exposed individuals enabling accurate determination of seroconversion. The developed assay is currently applied to evaluate immune responses to the Israeli vaccine during human phase I/II trials.

## INTRODUCTION

The first COVID-19 patient in Israel was identified on February 21st, 2020. By the end of April 2020, more than 15,000 people were verified as COVID-19 carriers/patients (https://www.worldometers.info/coronavirus/country/Israel/). This initial breakout was followed by a second (June to October 2020) and a third (December 2020 to March 2021) breakout, resulting in over 800,000 infected individuals and more than 6,000 deaths by the end of April 2021. During these months, authorities all over the world expressed the hope that exposure rates would result in the development of herd immunity, thus reducing disease spread. Due to the existence of asymptomatic individuals, which are estimated as 20% to 80% of total infections (1), actual exposure rates to SARS-CoV-2 are believed to be higher than the diagnosed reverse transcription-quantitative PCR (qRT-PCR) cases (2). Thus, the major tool for assessing the true seroprevalence and seroconversion within the population during the initial stages of the disease is serological testing. These tests determine the IgG/IgM response of the human adaptive immune system as a result of SARS-CoV-2 exposure and are thus the true indicators for disease spread.

At the beginning of the COVID-19 pandemic, as a prelude to a comprehensive serological survey of the Israeli population, a nationwide comparison of seven commercial serological assays was carried out by the Israeli Ministry of Health (3). The seven appraised commercial tests determined IgG signals mostly on one antigen (spike-based or nucleocapsid [NC]). The results demonstrated considerable variation among the tests, where apparent slight differences in specificity had profound effects on the expected positive predictive values (PPV) of each test. The follow-up survey, carried out by the Ministry of Health between July and September 2020 (*n* = 55,000), indicated that 5.5% of the Israeli population underwent seroconversion (4). These results concur with data collected in other countries (5 to 20% seroconversion) after COVID-19’s initial waves (5–7). However, a recent perspective paper (1) analyzing several serological surveys argues that some of them might not portray accurate results. This is due, in part, to employing a single antigen to determine seroconversion. Thus, antibodies from previous exposure to circulating coronaviruses may cross-react with the spike or the NC (8, 9), leading to overestimation of exposure.

The aim of the present work was therefore 2-fold: (i) development of a sensitive, specific serological tool that will enable discrimination between naive and exposed individuals, applicable for further characterization of different populations during the pandemic as well as during vaccination campaigns, and (ii) generation of a serological signature of the Israeli adult population at the beginning of the COVID-19 pandemic, an effort that joins similar works carried out by many countries worldwide (5, 6, 10–13). To this end, we developed a 6-plex antigen microarray using the whole inactivated virus, a recombinant NC protein, and four spike-derived recombinant antigens, including a stabilized version of SARS-CoV-2 spike ectodomain (S2P), the S1 subunit, N-terminal domain (NTD), and receptor-binding-domain (RBD). Assay format enabled simultaneous detection of IgG, IgM, and IgA in a single sample. This methodology has recently been applied for serological detection of SARS-CoV-2 patients by the assembly of both spike and nucleocapsid (NC) antigens to improve specificity (14–16). We hypothesized that this strategy (epitope multiplication) will allow the generation of a specific, reliable serological signature of COVID-19-exposed individuals.

This array was applied for diagnosis of SARS-CoV-2 seroconversion in 55 qRT-PCR-positive symptomatic/convalescent patients. qRT-PCR-negative subjects (*n* = 146), collected in Israel during the first 2 months of the pandemic, were used to generate a set of distinguishing parameters for naive versus symptomatic/asymptomatic individuals for each of the antigens incorporated in the array and capture a serological snapshot of the early-pandemic Israeli adult population.

## RESULTS

### Sample collection.

**FIG 1:**
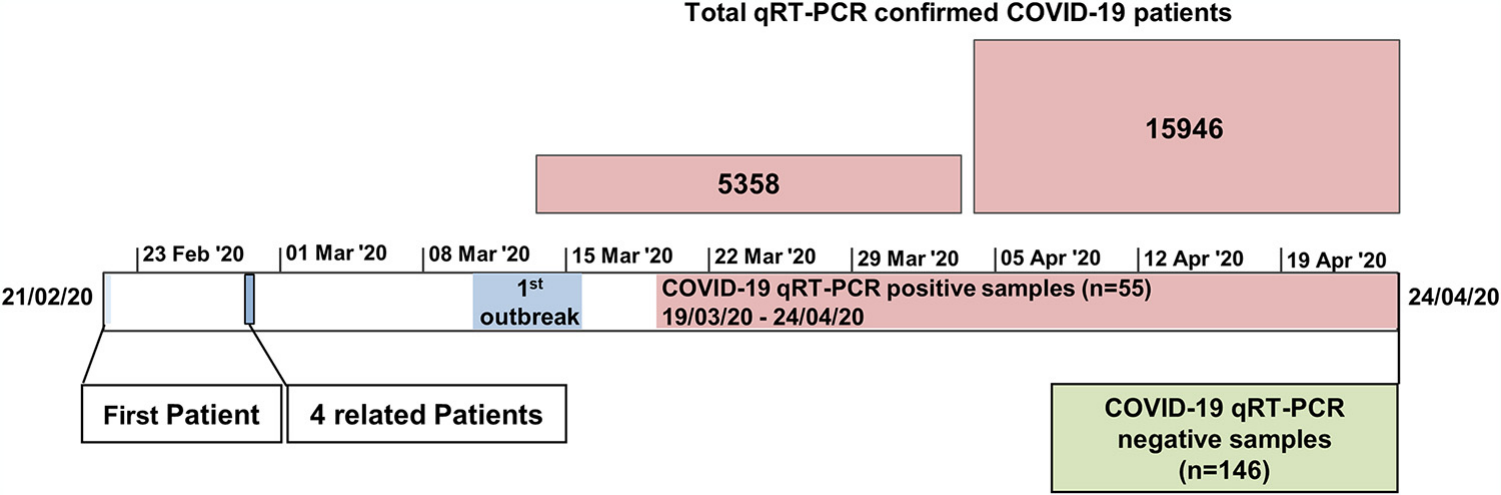
Timeline of COVID-19 pandemic initiation in Israel, relative to sample collection. Pink bars represent the total qRT-PCR confirmed samples in Israel in March and April 2020.

The first COVID-19 patient was identified in Israel on February 21st, and by the end of April 2020 there were 15,946 verified COVID-19 cases (Fig. 1). Between mid-March and the end of April, we obtained blood samples from 55 confirmed patients from a corona ward at Sheba hospital and from quarantine facilities in Israel. In addition, by the end of April, we acquired 146 blood samples from qRT-PCR-negative volunteers from all over the country. Since at that point COVID-19 was relatively contained and rare, these PCR-negative samples possibly represent the serological state of the adult population in Israel pre-COVID-19. Available demographic details pertaining to COVID-19 patients and qRT-PCR-negative donors are listed in Table 1.

**TABLE 1:** Demographics of qRT-PCR-positive and -negative individuals

	SARS-CoV-2 qRT-PCR-positive (*n* = 55)		SARS-CoV-2 qRT-PCR-negative (*n* = 146)	
Demographic	*n*	%	*n*	%
Age (yrs)				
20–30	16	29	6	4
31–40	10	18	24	16
41–50	4	7	43	29
51–60	7	13	48	33
61–70	7	13	26	18
>71	11	20		
Avg age	48.1		44.2	
Gender				
Male	32	58	74	51
Female	23	42	72	49
Sickness severity				
Mild	41	74.5		
Moderate	8	14.5		
Severe	**5**	9.1		
Critical	**1**	1.8		

### Reactivity of naive and SARS-CoV-2 patients/convalescent-phase sera with SARS-CoV-2 antigens.

The host response to viral exposure includes the accumulation of specific antibodies, i.e., seroconversion. The presence of SARS-CoV-2-specific IgG, IgM, and IgA antibodies was evaluated using a protein microarray spotted with six viral antigens: the whole inactivated SARS-CoV-2 virus, the NC, and four spike-derived antigens, S2P, S1, RBD, and NTD. The selected spike-derived antigens appear redundant, as the S2P protein contains the S1 unit, which in turn contains both the RBD and the NTD. As sequence homology between SARS-CoV-2 and other coronaviruses is high throughout the spike protein but lower in the RBD and NTD (17), this epitope combination was predicted to differentiate between nonspecific reactions and true COVID-19-related seroconversion. The whole virus, spotted on the array, has the added value of capturing antibodies directed against additional nonspike outer proteins (membrane and envelope structural proteins).

**FIG 2:**
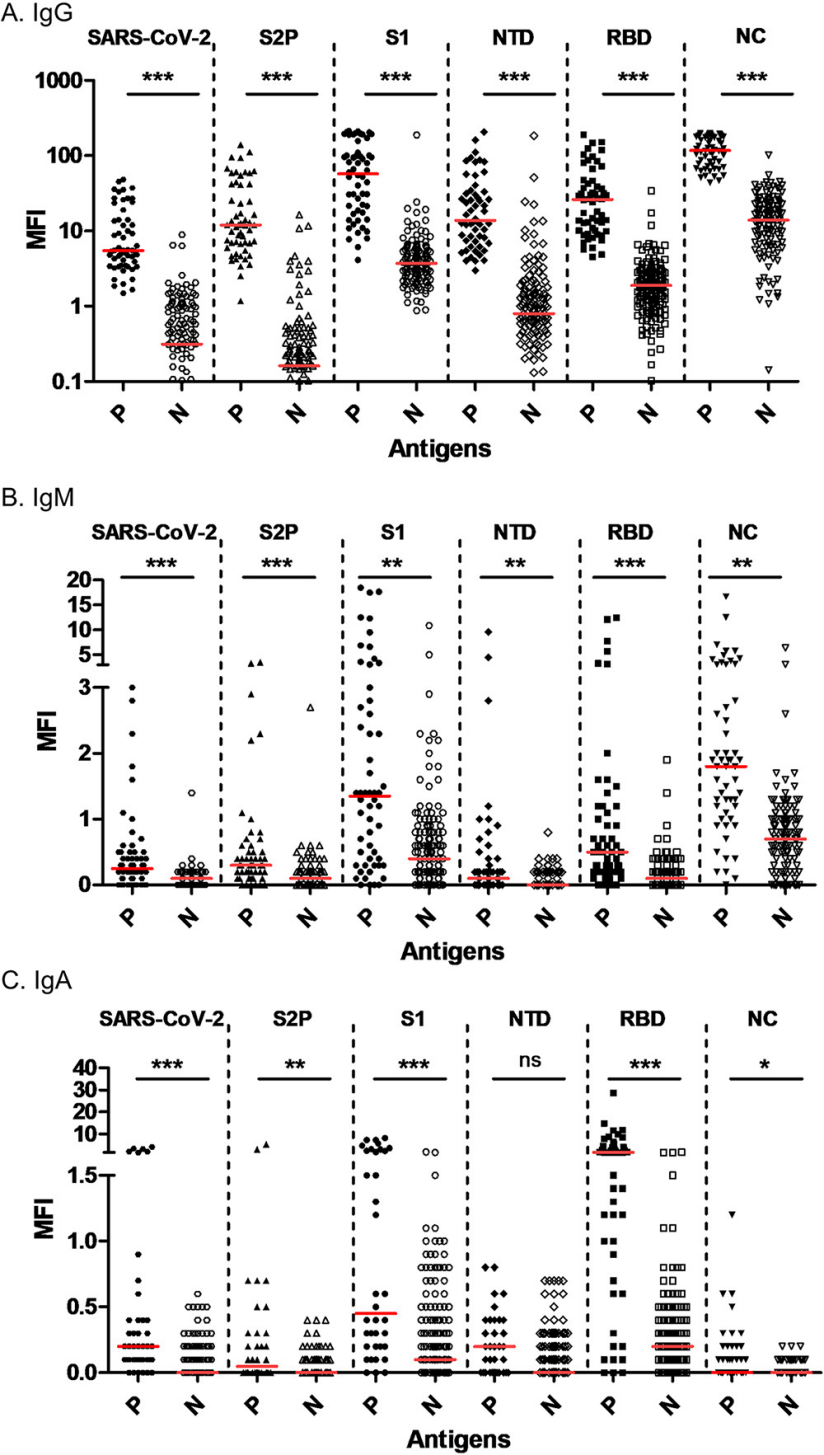
Scatterplot of MFI values of qRT-PCR-positive and -negative sera on the 6-plex SARS-CoV-2 protein array. (A) IgG, (B) IgM, and (C) IgA signals of qRT-PCR-positive (P; closed shapes) and -negative (N; open shapes) sera, analyzed on various SARS-CoV-2 proteins: inactivated SARS-CoV-2 virus (hexagons), S2P (triangles), S1 (circles), NTD (diamonds), RBD (squares), and NC (upside-down triangles). Each serum was evaluated against the 6-plex array. The distribution of the signals obtained from individual positive/negative serum samples is presented for each antigen. Horizontal red lines indicate the median value for each set. Statistical analysis was performed using one-way ANOVA followed by Dunn’s multiple-comparison test, using GraphPad Prism 6. ***, *P* < 0.001; **, *P* < 0.01; *, *P* < 0.05; ns, not significant.

To determine the optimal parameters that will enable differentiation between naive and SARS-CoV-2-exposed individuals, qRT-PCR-positive (*n* = 55) and -negative (*n* = 146) samples (Table 1) were analyzed for immunoglobulin level (IgG, IgM, and IgA). Scatterplots of individual mean fluorescent intensity (MFI) values for IgG, IgM, and IgA for positive (P) versus negative (N) sera versus all six antigens are presented in Figure 2. Results are presented as MFI, and the binding of each individual’s serum antibodies (IgG, IgM, and IgA) to all the spotted antigens is obtained simultaneously from a single subarray. Significant fluorescent signals were obtained with positive sera samples for all the tested immunoglobulins (IgG, IgM, and IgA) on the 6-plex antigen panel. As indicated, the differences between P and N samples for all six antigens were statistically significant for both IgG and IgM (Fig. 2A and B). For IgA, the differences for all but NTD were significant, as well (Fig. 2C).

### Biomarkers classification.

By measuring multiple biomarkers in numerous individuals, it is possible to create a characteristic disease-specific serological signature. The accuracy of the resulting signature depends strictly on the ability to differentiate between infected and uninfected individuals, enabling high detection sensitivity as well as high specificity. We investigated the tradeoff between these two parameters by varying the cutoff MFI value for seropositivity/negativity using receiver operation characteristic (ROC) curves. ROC curves are created by plotting true-positive rates against false-positive rates at various threshold settings. An antigen, classified as the ultimate predictor, would yield a point in the upper left corner of the ROC space, representing 100% sensitivity and 100% specificity. In real-life scenarios, cutoff values are chosen as a compromise between sensitivity and specificity. ROC curves (Fig. S1) were determined for each individual antigen for all antibody serotypes (IgG, IgM, and IgA) using the MFI values presented in Figure 2. The selected threshold values (highlighted in bold), areas under the curve (AUC), and the sensitivity and specificity for each antigen at the chosen cutoff values are summarized in Table S1. It is evident that IgG signals (Fig. S1A), unlike IgM and IgA signals (Fig. S1B and C), enable optimum detection, lowering both false-negative (FN) and false-positive (FP) rates. This is supported (Table S1) by the higher AUC values (0.97 to 0.99, indicating high diagnostic accuracy) that were obtained with IgG for all six antigens, resulting in high sensitivity (91% to 100%) and specificity (84% to 98%) at the determined cutoff values. It appears that NC is the best parameter (Fig. S1, purple line), demonstrating 100% sensitivity and 95.2% specificity for IgG (Table S1).

**FIG 3:**
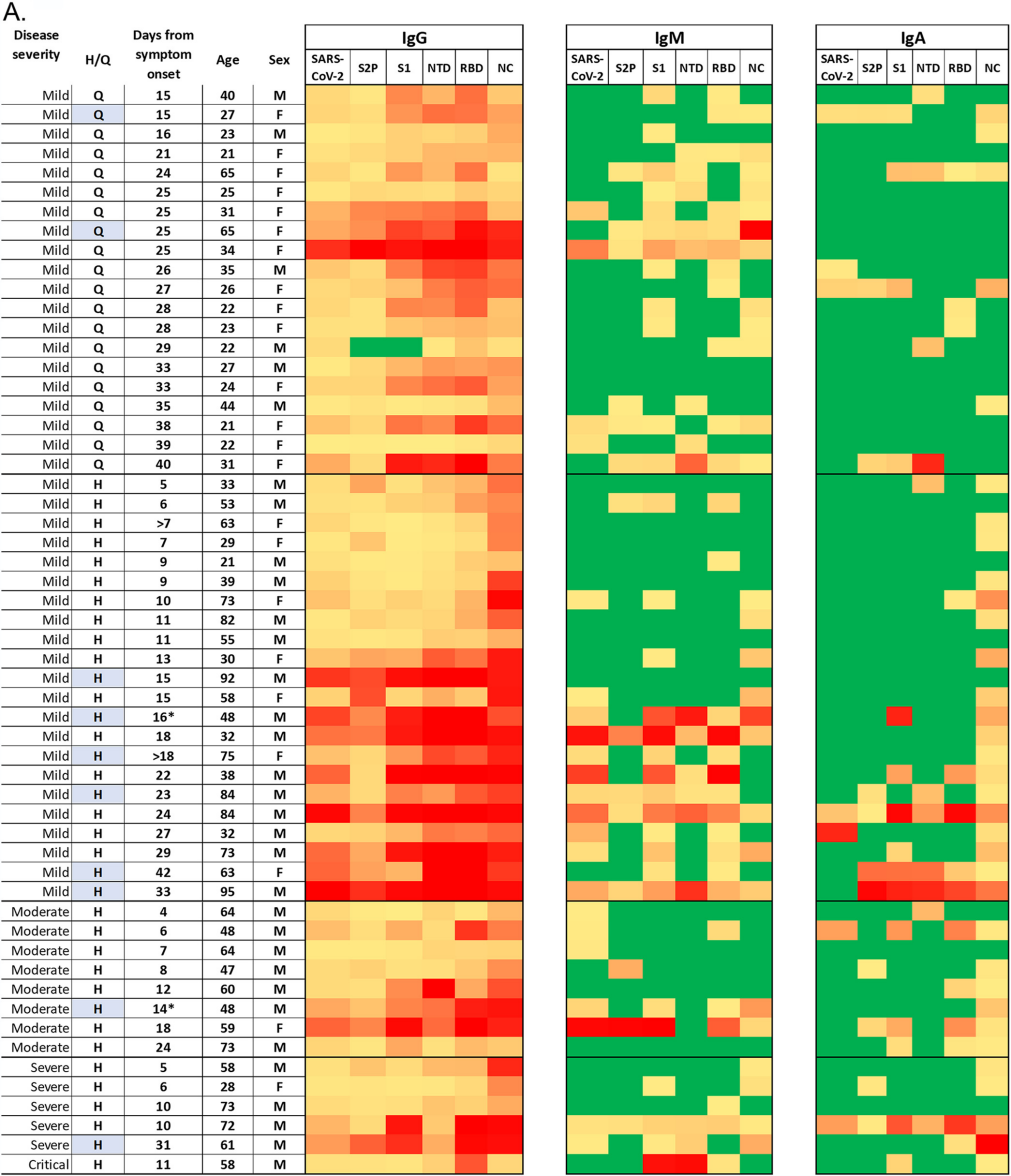

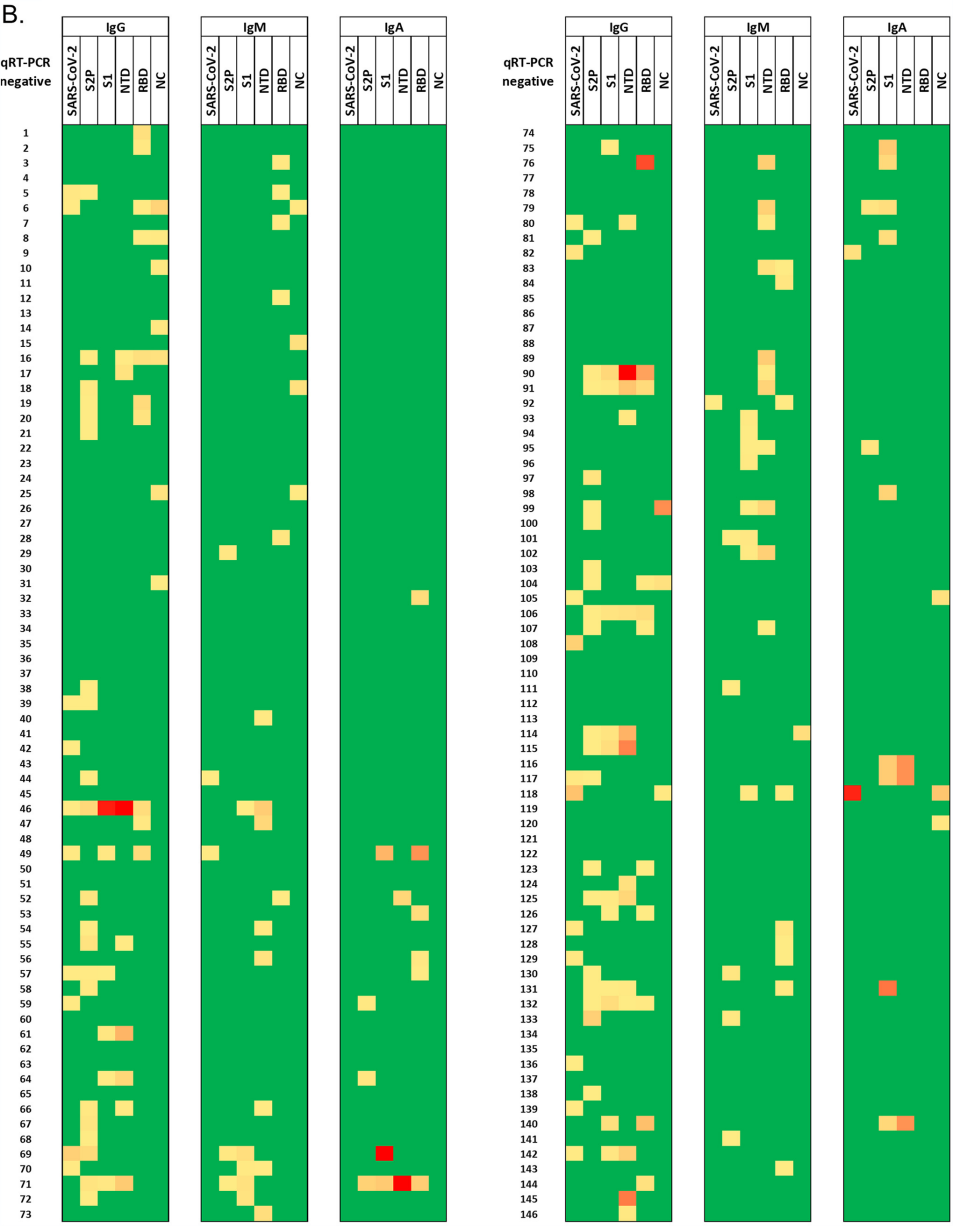
Heat maps of qRT-PCR-positive and -negative individuals tested with SARS-CoV-2 6-plex array. Individual sera of (A) qRT-PCR-positive and (B) qRT-PCR-negative individuals were incubated with the 6-plex protein array. MFI signals are presented after subtraction of the previously determined cutoff values for each antigen (Table S1). Results are presented for all antibody isotypes, left to right: IgG, IgM, and IgA on inactivated SARS-CoV-2 and recombinant antigens S2P, S1, NTD, RBD, and NC. qRT-PCR-positive individuals are presented according to disease severity (mild, moderate, severe, and critical), hospitalized (H), or quarantined (Q), 4 to 40 days from symptom onset. Colors: yellow to red, MFI values from low to high; green, no interaction; blue, patients with known background conditions. *, this patient was analyzed at two different time points.

To generate the serological signature of each of the tested individuals at the established settings, heat maps of qRT-PCR-positive (Fig. 3A) and qRT-PCR-negative (Fig. 3B) individuals were generated, portraying the MFI signals of each individual on all antigens for all antibody isotypes after subtraction of each antigen’s specific cutoff value (highlighted in bold in Table S1).

Analysis of qRT-PCR-positive individuals (Fig. 3A) indicates that all but one of the samples present full IgG-specific seroconversion on all six antigens presented in the microarray. In contrast, IgM and IgA react simultaneously with only a maximum of 3/6 antigens, and in some cases with none, and are thus not suitable as seroconversion indicators. Seroconversion rate (sensitivity), determined as a reaction with the full antigen panel, was 98.2%, 8.9%, and 3.6%, for IgG, IgM, and IgA, respectively (Fig. 3A).

Examination of the heat map generated from the qRT-PCR-negative subjects (Fig. 3B) immediately highlights the benefits of using a 6-plex antigen panel for discerning between naive and exposed individuals, as despite some nonspecific interactions present in the qRT-PCR-negative population, none of the samples (0/146) reacted with all six antigens (100% specificity). IgG signals exhibited the highest rate of nonspecific reactions, with 1/146 (0.7%) reacting with five of the antigens, followed by 5/146 (3.4%), 10/146 (6.8%), 18/146 (12.3%), 42/146 (28.8%), and 70/146 (47.9%) that reacted with 4, 3, 2, 1, or none of the antigens, respectively.

## DISCUSSION

**FIG 4:**
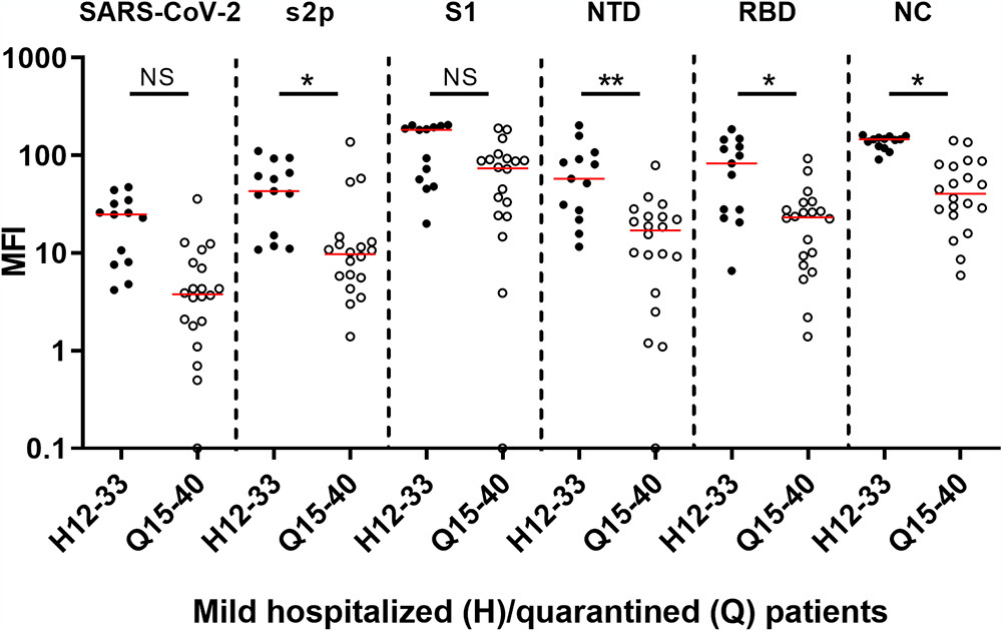
Scatterplot of MFI values of IgG antibodies present in mild hospitalized versus mild quarantined sera, 13 days from symptom onset. MFI values (Fig. 3A) of IgG antibodies present in sera of mild hospitalized patients, 13 to 33 days after symptom onset (H12-33, closed circles) were compared to those from mild quarantined patients, 15 to 40 days after symptom onset (Q15-40, open circles) on the 6-plex array (antigens are indicated at the top of the panel). Horizontal red lines indicate the median value for each set. Statistical analysis was performed using one-way ANOVA followed by Dunn’s multiple-comparison test, using GraphPad Prism 6. **, *P* < 0.01; *, *P* < 0.05; ns, not significant.

In this work, we used a 6-plex antigen array, presenting five viral proteins and the whole SARS-CoV-2 virus, to determine specific seroconversion in SARS-CoV-2 qRT-PCR-positive and -negative subjects. We found that antibody responses, generated following SARS-CoV-2 exposure, were diverse with respect to antibody isotypes, presenting different patterns on the 6-plex array (Fig. 3A). Of the three antibody isotypes, IgG signals were the best indicators of seroconversion, with all but one individual presenting positive IgG signals on all of the array’s presented antigens (Fig. 3A). Analysis of the qRT-PCR-positive samples indicated that seroconversion can be detected in hospitalized patients presenting mild symptoms as soon as 5 days after symptom onset, with signals on NC being the best indicators for seroconversion. Within those patients (mild, hospitalized), we found a positive correlation between signal intensity and time from symptom onset, with enhanced signals starting on day 13. Interestingly, this correlation was not observed in mildly ill quarantined subjects, which generally maintained lower IgG levels throughout their disease course. This difference is statistically significant against S2P, NTD, RBD, and NC (Fig. 4) and could presumably be explained by the fact that hospitalized patients present symptoms while quarantined subjects might be asymptomatic. Another significant difference (*P* = 0.01) between the same populations (mild hospitalized and mild quarantined) can also be seen in IgA values developed against NC (Fig. 3A, right panel, NC antigen and Fig. S2). This observed increase in IgA signals may indicate the development of IgA antibodies in the lung mucosal surface (1), which may point to the existence of breathing/respiratory difficulties that manifest in hospitalized but not quarantined individuals. Previous studies reported a correlation between disease severity and IgA signals developed on RBD (18) and S2P (19), a difference that was not significant in our study. Other factors that might be responsible for the differences observed between quarantined and hospitalized individuals are age and sex, as the average age of the quarantined individuals was 31 (*n* = 20) and the individuals were predominantly female (68.2%), while the average age for hospitalized mild, moderate, and severe patients was 56.9 (*n* = 22), 57.9 (*n* = 8), and 58.3 (*n* = 6) and the individuals were predominantly male (68.2%, 87.5%, and 83.3%). Overall, seroconversion in qRT-PCR-positive subjects could be determined by IgG levels developed against the two main SARS-CoV-2 proteins: spike and NC. These results concur with the ability of NC or spike-based commercial tests to accurately identify seroconversion in patients and convalescent individuals.

This, however, is not the case for the qRT-PCR-negative subjects (Fig. 3B), where reaction profiles varied, probably due to nonspecific interactions that may arise from previous exposure to seasonal coronaviruses (12, 20). Results indicate that unlike the IgG pattern observed for the qRT-PCR-positive samples (Fig. 3A), a reaction with one of the spike-derived antigens was not always accompanied by a reaction with the other spike-derived antigens (Fig. 3B). Positive IgG signals against at least one of the four spike-derived antigens were present in 61/146 (42%) of the samples (Fig. 3B). Only 5/146 samples (3.4%) presented positive reactions with all four spike-derived antigens, while additional samples (5/146) reacted with S2P, S1, and NTD but not with RBD. Reaction with both spike-derived and NC antigens was detected in only 5/146 samples, one of which reacted with the NC and a total of three spike-derived proteins (S2P, NTD, and RBD). Surprisingly, in four of these cases, the spike-derived antigen was RBD, which is considered highly specific to SARS-CoV-2. This positive interaction is hypothetically directed against less-specific epitopes in the RBD, common with other coronaviruses.

Out of the tested antigens, NC conferred the highest specificity compared to the other tested antigens (Fig. 3B and Table 2), with low signals detected in 10/146 (6%) of the samples. This result is somewhat unexpected, as NC is quite conserved in many coronaviruses (21, 22). The only naive individual displaying a positive signal with five of the analyzed antigens did not react with the NC, indicating that the NC antigen and at least one spike-based antigen are crucial for unequivocal discrimination between naive and exposed individuals.

**TABLE 2:** Antigen specificity in the qRT-PCR-negative cohort

Antigen	SARS-CoV-2	S1	S2P	RBD	NTD	NC	All^*a*^
False positive	21/146	18/146	41/146	21/146	22/146	10/146	0/146
Specificity	86%	88%	72%	86%	85%	93%	100%

a Reaction with the full antigen panel.

Based on IgG signals, analysis of a single biomarker for specificity classification (as applied by most commercial lateral flow assays) results in a specificity range of 75.0% to 94.6% (Table 2), with NC as the most specific indicator and S2P as the least specific. Our findings indicate that 75/146 (45%) of the adult Israeli naive qRT-PCR-negative cohort had nonspecific interactions with one or more SARS-CoV-2 antigens while none reacted with all of them. We therefore claim that the determination of seroconversion based on one antigen is not accurate and might lead to overestimation of the actual seroconversion in the population. A serological signature based on antibody responses to multiple antigens, such as the one demonstrated in this study, provides a more accurate and robust classification of individuals that were exposed to SARS-CoV-2. Evidence as to whether these cross-reactions confer protection against SARS-CoV-2 infection is conflicting, with some studies indicating that these reactions are not correlated with protection (3, 20) and others claiming that they are the cause of the different disease progressions and outcomes observed in some regions of the world (12, 23).

Our 6-plex assay demonstrated 98.2% sensitivity, 100% specificity, and a positive predictive value and a negative predictive value of 1.000 (0.9352 to 1.000) and 0.9939 (0.9667 to 0.9998), respectively. Based on the cohort of 146 randomized healthy individuals, we found zero prevalence (reaction with all six antigens presented on the microarray) of anti-SARS-CoV-2 antibodies in the adult population of Israel during the first months of the COVID-19 pandemic. The results presented herein coincide with results presented by others, indicating low to no seroprevalence of anti-SARS-CoV-2 antibodies at the earlier stages of the pandemic in many countries around the globe (5, 6, 10–12). An actual comparison is complicated, as it depends on disease prevalence and time span of sample collection (1), as well as the serological assay employed. For example, volunteers’ sera examined in Greece (10), the Netherlands (13), and some parts of the United States (5) until the end of April 2020 demonstrated antibody prevalence of 0.36%, 2.7%, and 1 to 6.9%, respectively, whereas sera collected at later stages (May to June 2020) in Switzerland and Kenya revealed 4.8% and 5.6% prevalence, respectively (6, 11). It is important to point out that since most of the data obtained in the above-cited works were obtained using commercial kits employing one antigen, a possible overestimation of the seroconverted population is likely.

The presented microarray was used as a basis for the design of an advanced array that is now being used to evaluate immune responses to the Israeli vaccine during human phase I/II trials.

## MATERIALS AND METHODS

### Antigens.

SARS-CoV-2 recombinant proteins RBD, NTD, S1, S2P, and NC were designed, expressed, and purified as described in detail previously (24–26). SARS-CoV-2 (GISAID accession EPI_ISL_406862) was kindly provided by Bundeswehr Institute of Microbiology, Munich, Germany and cultivated on VERO E6 cells as described previously (27). For array spotting, the virus was inactivated by a 48-h incubation with β-propiolactone (βpL) (SERVA electrophoresis GmbH, Heidelberg, Germany) diluted to a final concentration of 1:500.

### Clinical samples.

Serum samples were obtained from qRT-PCR-positive quarantined patients (*n* = 20, mild to no symptoms) and from hospitalized patients (*n* = 35, mild to severe condition, Shiba hospital, Tel-HaShomer, Israel). Samples were collected 4 to 40 days from symptom onset. Serum samples from qRT-PCR-negative volunteers (*n* = 146) were collected on April 24, 2020. Commercial normal human serum (human pooled serum, catalog no. 2931149, lot Q8441, MP Biomedicals LLC) was used as a prepandemic control. All samples were heat inactivated (56°C, 30 min) and stored at −20°C prior to testing.

### Serological assays.

Inactivated SARS-CoV-2 virus (2 × 10^8^ PFU/ml) as well as five purified SARS-CoV-2 antigens, including S2P, RBD, NTD, S1, and NC (0.2 to 1 mg/ml), was spotted (2 drops of 300 pl) in sextuplicate on nitrocellulose-coated slides containing 16 subarrays (Grace Bio Labs, GBL, Bend, OR) using a noncontact Piezo dispensing microarray spotter (Scienion Inc.). Slides were blocked with array buffer (2% gelatin, 0.1% Tween 20, 3.3% bovine serum albumin [BSA] in phosphate-buffered saline [PBS]) for 30 min at room temperature, washed (0.1% Tween 20 in PBS), dried, and stored desiccated until use. Diluted sera (1:500 in array buffer) were loaded on the slides. Incubations were carried out for 30 min at room temperature on a plate shaker. Following incubation, the slides were washed thrice (0.1% Tween 20 in PBS) and incubated with a mixture of three secondary reporter antibodies: Alexa Fluor 647 goat-anti-human IgG (H+L; ThermoFisher Scientific, A21445), Alexa Fluor 488 AffiniPure goat-anti-human IgM, Fc_5μ_ fragment specific (Jackson ImmunoResearch Laboratories, 109-545-043), and Alexa Fluor 532 AffiniPure goat-anti-human serum IgA, α chain specific (Jackson ImmunoResearch Laboratories, 109-005-011), diluted (1:300) in array buffer. The anti-human IgA antibody was conjugated to Alexa Fluor 532 (Alexa Fluor 532 protein labeling kit, Thermo Fisher Scientific, A10236) according to the manufacturer’s instructions. The slides were washed again and dried. Slides were scanned using a SciReader FL2 system (Scienion, Germany) and the mean fluorescence intensity (MFI) for each spot was recorded using scanArray software.

### Statistical evaluation of diagnostic performance.

The mean fluorescence of six replicates for each antigen was used for statistical analysis. The MFI values of the nonspecific interaction of the secondary antibodies, generated using a PBS control, were subtracted from all signals. Analysis of the significance of the differences between qRT-PCR-positive and -negative individuals was carried out using one-way analysis of variance (ANOVA), followed by Dunn’s multiple-comparison test applying GraphPad Prism 6 (La Jolla, CA). Sensitivity and specificity were determined by receiver operating characteristic (ROC) curves and area under the curve (AUC) calculations, computed with the same software. Delong method was used for calculating AUC confidence intervals. Cutoff values for positive versus negative results were set to a maximum Youden’s index (28).

### Ethics statement.

Sample collection was approved by the SMC institutional review board committee for broad antibody testing (approval number 7036-20-SMC). The experiment was approved by the Israeli Health Ministry (reference number 176942220). All patients gave their written informed consent before the examination was performed. The relevant regulations and institutional polices were followed strictly.
