# Supplementary material for: A Serological Snapshot of COVID-19 Initial Stages in Israel by a 6-Plex Antigen Array
Source: Microbiol Spectr. 2021 Oct 6;9(2):e00870-21. doi: 10.1128/Spectrum.00870-21 (PMC8510178; doi:10.1128/Spectrum.00870-21)
Supplement: SUPPLEMENTAL FILE 1 — Supplemental material. Download SPECTRUM00870-21_Supp_1_seq8.pdf, PDF file, 0.3 MB [file spectrum00870-21_supp_1_seq8.pdf]

**Table S1: Results of ROC curve analysis for the six array's antigens**

| | IgG | | | | IgM | | | | IgA | | | |
|---|---|---|---|---|---|---|---|---|---|---|---|---|
| | **AUC** (95% CI) | **Cut Off** (MFI) | **Sensitivity** | **Specificity** | **AUC** (95% CI) | **Cut Off** (MFI) | **Sensitivity** | **Specificity** | **AUC** (95% CI) | **Cut Off** (MFI) | **Sensitivity** | **Specificity** |
| **S1** | 0.971 (0.94-0.99) | **7** | 96.1% | 84.2% | 0.730 (0.65-0.83) | **1.2** | 53.1% | 91.2% | 0.744 (0.65-0.83) | **0.8** | 41.6% | 91.1% |
| **s2p** | 0.985 (0.97-0.99) | **1.1** | 98.2% | 90.6% | 0.677 (0.58-0.76) | **0.3** | 25.1% | 93.2% | 0.677 (0.56-0.79) | **0.2** | 27.1% | 96.2% |
| **NTD** | 0.959 (0.93-0.98) | **2.9** | 98.2% | 86.3% | 0.778 (0.70-0.85) | **0.4** | 41.3% | 91.4% | 0.636 (0.53-0.74) | **0.3** | 37.2% | 80.1% |
| **RBD** | 0.987 (0.94-0.99) | **4.4** | 98.2% | 91.8% | 0.790 (0.71-0.86) | **0.5** | 52.2% | 94.3% | 0.752 (0.67-0.83) | **0.3** | 40.1% | 89.2% |
| **NC** | 0.996 (0.99-1.00) | **40.0** | 100.0% | 95.2% | 0.825 (0.75-0.89) | **1.8** | 52.3% | 98.1% | 0.850 (0.77-0.93) | **0.9** | 74.4% | 95.8% |
| **SARS-CoV-2** | 0.984 (0.97-0.99) | **1.2** | 91.3% | 98.1% | 0.712 (0.62-0.81) | **0.4** | 39.0% | 96.0% | 0.648 (0.54-0.74) | **0.2** | 26.6% | 97.1% |

AUC- area under the curve diagnostic ability: <0.7: not accurate; 0.7-0.9: moderately accurate; >0.9: highly accurate. CI – confidence intervals.

Cutoff values (MFI) for each antibody isotype on each antigen are highlighted in bold

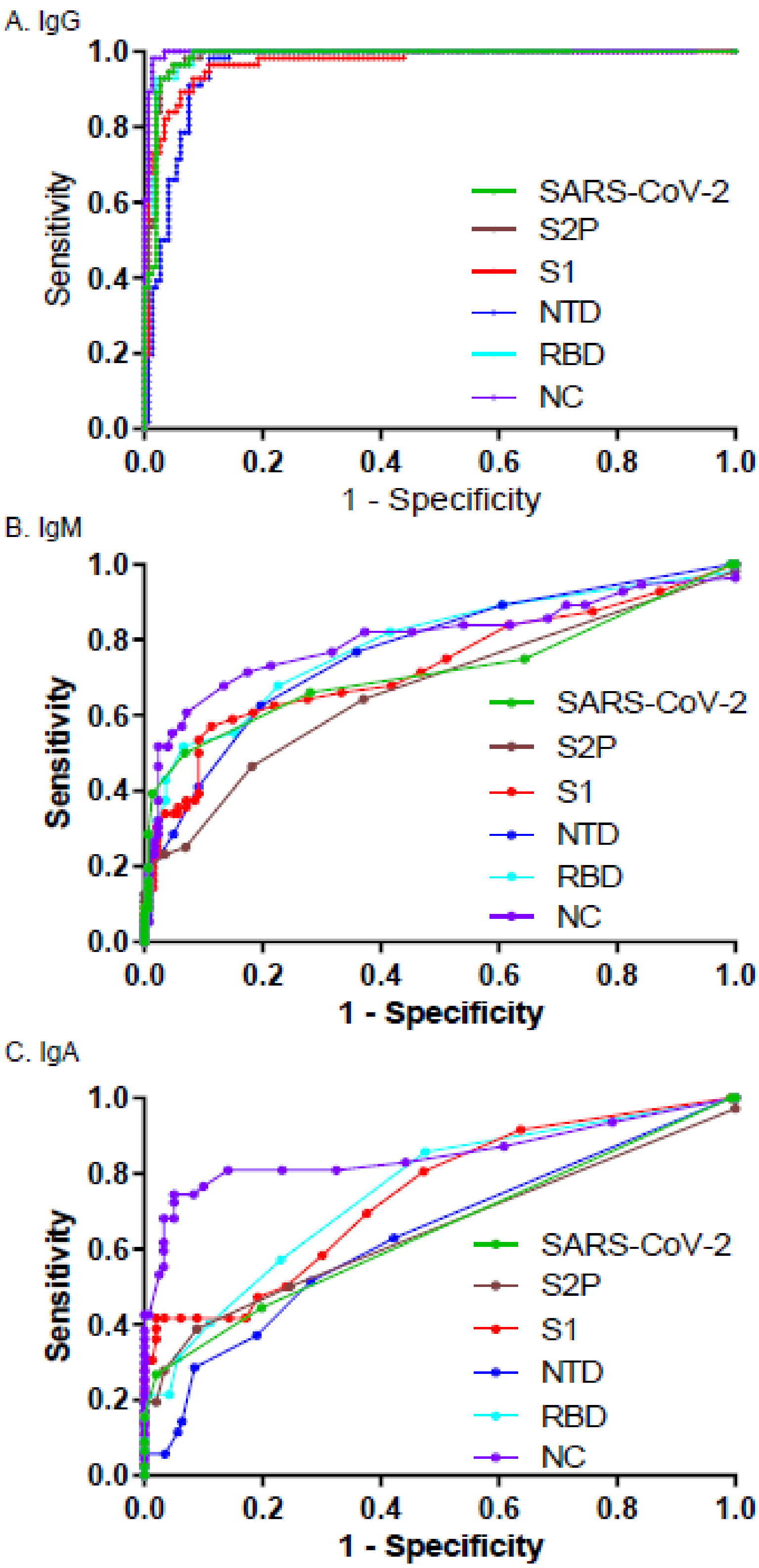


**Fig S1**. **ROC analysis.** ROC curves for A. IgG antibodies, B. IgM antibodies and C. IgA antibodies against inactivated SARS-CoV-2 (green), S2P (brown), S1 (red), NTD (blue), RBD (cyan) and NC (purple), were generated from crude MFI values (minus reagents background MFI values) obtained for qRT-PCR positive and negative sera samples shown in Fig 2.

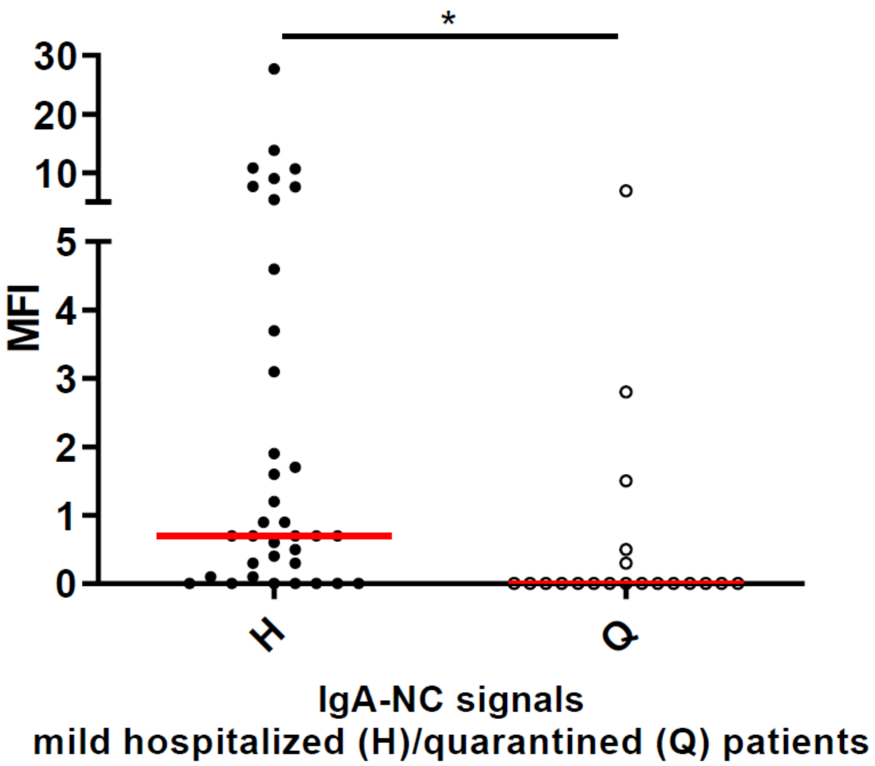


**Figure S2**. **Scatter plot of MFI values of anti-NC IgA antibodies present in mild hospitalized vs mild quarantined sera.** MFI values (Fig. 3A) of IgA antibodies against NC present in sera of mild hospitalized patients (H, closed circles) were compared to those from mild quarantined patients (Q, open circles). Horizontal red lines indicate the median value for each set. Statistical analysis was preformed using T-Test (GraphPad Prism 6). * p-value < 0.05
